# Salidroside Ameliorates Mitochondria-Dependent Neuronal Apoptosis after Spinal Cord Ischemia-Reperfusion Injury Partially through Inhibiting Oxidative Stress and Promoting Mitophagy

**DOI:** 10.1155/2020/3549704

**Published:** 2020-07-23

**Authors:** Changjiang Gu, Linwei Li, Yifan Huang, Dingfei Qian, Wei Liu, Chengliang Zhang, Yongjun Luo, Zheng Zhou, Fanqi Kong, Xuan Zhao, Hao Liu, Peng Gao, Jian Chen, Guoyong Yin

**Affiliations:** ^1^Department of Orthopedics, The First Affiliated Hospital of Nanjing Medical University, Nanjing, Jiangsu Province, China; ^2^Department of Orthopedics, The People's Hospital of Shuyang City, Jiangsu Province, China

## Abstract

Ischemia-reperfusion injury is the second most common injury of the spinal cord and has the risk of neurological dysfunction and paralysis, which can seriously affect patient quality of life. Salidroside (Sal) is an active ingredient extracted from Herba Cistanche with a variety of biological attributes such as antioxidant, antiapoptotic, and neuroprotective activities. Moreover, Sal has shown a protective effect in ischemia-reperfusion injury of the liver, heart, and brain, but its effect in ischemia-reperfusion injury of the spinal cord has not been elucidated. Here, we demonstrated for the first time that Sal pretreatment can significantly improve functional recovery in mice after spinal cord ischemia-reperfusion injury and significantly inhibit the apoptosis of neurons both *in vivo* and *in vitro*. Neurons have a high metabolic rate, and consequently, mitochondria, as the main energy-supplying suborganelles, become the main injury site of spinal cord ischemia-reperfusion injury. Mitochondrial pathway-dependent neuronal apoptosis is increasingly confirmed by researchers; therefore, Sal's effect on mitochondria naturally attracted our attention. By means of a range of experiments both *in viv*o and *in vitro*, we found that Sal can reduce reactive oxygen species production through antioxidant stress to reduce mitochondrial permeability and mitochondrial damage, and it can also enhance the PINK1-Parkin signaling pathway and promote mitophagy to eliminate damaged mitochondria. In conclusion, our results show that Sal is beneficial to the protection of spinal cord neurons after ischemia-reperfusion injury, mainly by reducing apoptosis associated with the mitochondrial-dependent pathway, among which Sal's antioxidant and autophagy-promoting properties play an important role.

## 1. Introduction

Clinically, thoracoabdominal aortic operations or decompression surgery of the spinal cord often lead to ischemia-reperfusion injury [1, 2], generating neurologic dysfunction, paralysis of lower limbs, and other unpredictable disastrous consequences, which puts a huge burden on patients and society [3, 4]. Although considerable therapeutic interventions have been proposed to alleviate the damage of neurological function after spinal cord ischemia-reperfusion injury (SCIRI), the overall effect is limited.

Although diverse pathophysiologic mechanisms of SCIRI have been proposed, it is widely considered that neuronal death is induced by apoptosis [5, 6]. Apoptosis is triggered by an intrinsic and extrinsic pathway. The intrinsic pathway is mitochondrial centered [7]. On the one hand, alterations in mitochondrial function will result in an adenosine triphosphate level reduction, Ca^2+^ homeostasis damage, and reactive oxygen species (ROS) stress injury, and on the other hand, a series of proapoptotic substances, such as cytochrome C, will be released from the injured mitochondria into the cytoplasm to activate caspase-dependent apoptotic cascade events and eventually lead to neuron death [8]. Hence, it is crucial to maintain mitochondrial homeostasis for cell survival. Neurons are vulnerable to ischemic injury because of their high demand for energy [9]. Mitochondrial dysfunction has been recognized as the initial step of neuronal injury during spinal cord ischemia, and it is also crucial for the amplification of secondary injury and subsequent neuronal cell death associated with the increase in mitochondrial oxidative damage and prodeath proteins [10–12]. How to reduce mitochondrial injury and remove damaged mitochondria in a timely manner to maintain the homeostasis of the intracellular environment is one of the greatest challenges of SCIRI therapy.

ROS play crucial roles in the occurrence and development of ischemia-reperfusion (I/R) injury [13] and are closely related to mitochondrial homeostasis [14, 15]. Excessive ROS accumulation can induce mitochondrial cristae expansion, outer mitochondrial membrane rupture, and cytochrome C release, ultimately resulting in apoptosis [16]. In recent years, the reduction of neuronal apoptosis by antioxidants has been an important auxiliary means to prevent or treat SCIRI [17–19]. Researchers are also discovering powerful antioxidants to treat SCIRI with few side effects.

Autophagy is an evolutionarily conserved process in eukaryotes, involving the degradation and recycling of cytosolic, long-lived proteins and organelles [20]. The removal of damaged mitochondria through autophagy is known as mitophagy. Mitophagy is important for controlling the homeostasis of mitochondria and promoting cell survival [21]. In chronic neurodegenerative diseases or stroke, mitophagy has been found to be enhanced and beneficial [22, 23]. Recently, it was reported that promoting mitophagy during SCIRI can reduce neuronal apoptosis and restore nerve function [24, 25]. Therefore, we speculate that mitophagy plays a vital role in mitochondrial-dependent neuronal apoptosis during SCIRI.

**Figure 1:**
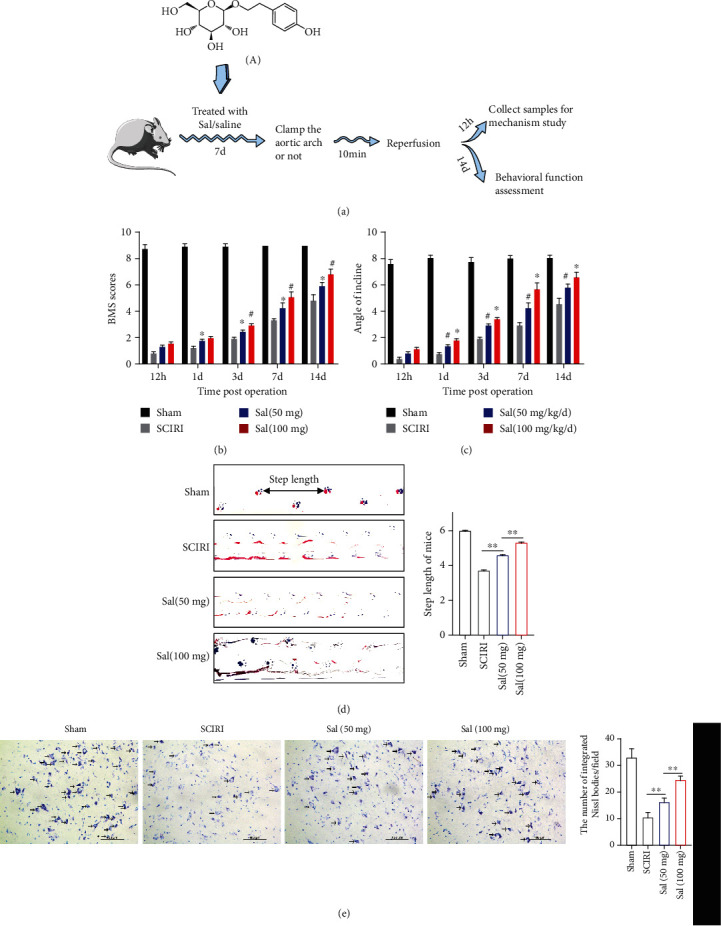
Sal promoted the recovery of motor function in mice and reduced the loss of motor neurons in the anterior horn after SCIRI. (a) Schematic diagram of the experimental design. (A) The chemical structure of Sal. (b) BMS scores at different time points following SCIRI. (c) The inclined plate test at different time points after SCIRI. (d) Representative paw prints at 14 days after SCIRI and quantitative analysis of step length of mice. Blue: paw print of the front paw; red: paw print of the hind paw. (e) Nissl staining indicating the number of motor neurons in the anterior horn of the spinal cord in each group. Scale bar, 100 *μ*m. ∗*p* < 0.05, compared to the SCIRI group; ^#^*p* < 0.05, compared with the Sal (50 mg/kg/d) group. All values in this figure were presented as mean ± SEM (*n* = 4), ∗∗*p* < 0.01.

Salidroside (Sal) (Figure 1(a), A) is a bioactive ingredient extracted from *Rhodiola rosea* L., possessing multiple pharmacological properties, such as antioxidative, anti-inflammatory, and antidiabetic activities [26–29]; it has also been reported to promote autophagy and to have a neuroprotective role [28, 30–32]. In recent years, a number of studies have explored the role of Sal in spinal cord injury and shown some protective effects. Wang et al. found that Sal regulated microglial autophagy flux through the AMPK/mTOR pathway, which then affected glial cell polarization and promoted functional recovery in rats after spinal cord injury [33]. Su et al. showed that Sal promotes rat spinal cord injury recovery by inhibiting inflammatory cytokine expression and NF-*κ*B and MAPK signaling in astrocytes [34]. The role of glial cells in the pathological process of spinal cord injury is indisputable. Improving the inflammatory microenvironment after spinal cord injury by affecting the function of glial cells is an important therapeutic target. However, it has not been reported whether Sal affects functional recovery after spinal cord injury through other mechanisms, especially for spinal cord neurons, which are the cells most affected by spinal cord injury. Emerging evidence has shown that Sal plays a protective role in I/R injury in the liver, heart, brain, and other systems [35–37]. However, its role in SCIRI is rarely reported. Hence, in this study, we studied the effect of Sal on spinal cord ischemia-reperfusion injury and its underlying mechanism.

## 2. Materials and Methods

### 2.1. Animals and Experimental Protocol

The experimental protocol was approved by the Ethical Committee of Nanjing Medical University, China, and all procedures were in accordance with the National Institutes of Health Guide for the Care and Use of Laboratory Animals. Male C57BL/6 mice (Animal Research Center of Nanjing Medical University, Nanjing, China) aged between 10 and 16 weeks were used for all experiments according to an established protocol. The SCIRI model was established as described previously [38]. In short, mice were anesthetized using 2% isoflurane and placed in the supine position. Heparin (170 IU/kg) was subcutaneously injected 5 min before the procedure. The aortic arch was exposed using a cervicothoracic approach as previously described [39]. Occlusion was achieved by placing vascular clamps (30 g forces, Oscar, Shanghai, China) on the aortic arch distal to the left common carotid artery and the subclavian artery. Ten minutes later, the clip was removed to restore perfusion. Bladders were manually expressed twice per day during the experimental period. A total of 84 mice were randomly assigned to four groups: the sham group, the SCIRI group, and the Sal treatment group. The Sal treatment group was further divided into high-dose (100 mg/kg/day) and low-dose (50 mg/kg/day) Sal treatment groups. The sham group was with 12 mice and the other three groups contained 36 mice, respectively. The mice in the Sal treatment group were injected intraperitoneally with Sal once a day for 7 days before the operation while the vehicle group was only given saline, after which both groups underwent SCIRI. In the sham group, only exposure of the aortic arch was performed, without clamping. After surgery, the animals in the Sal treatment group were immediately injected with Sal. The control group was injected with an equivalent dose of normal saline.

### 2.2. Antibodies and Reagents

The primary antibodies used were as follows: mouse anti-NeuN (ab104224, Abcam, Cambridge, United Kingdom); rabbit anti-cleaved caspase-9 (20750, Cell Signaling Technology, Danvers, MA, USA); rabbit anti-cleaved caspase-3 (9664, Cell Signaling Technology); rabbit anti-Bcl-2 (ab32124, Abcam); rabbit anti-Bax (ab32503, Abcam); rabbit anti-*β*-actin (4970, Cell Signaling Technology); rabbit anti-LAMP2 (ab13524, Abcam); rabbit anti-Tomm20 (ab186735, Abcam); rabbit anti-LC3B (ab48394, Abcam); mouse anti-P62 (ab56416, Abcam); mouse anti-PINK1 (ab186303, Abcam); mouse anti-Parkin (BC100-494, Novus Biologicals, Littleton, CO, USA); rabbit anti-phospho-ubiquitin (ser65; 62802, Cell Signaling Technology); mouse anti-Parkin (BC100-494, Novus); rabbit anti-VDAC (55259-1-AP, Proteintech, IL, USA); and rabbit anti-MAP2 (ab32454, Abcam). The secondary antibodies used were as follows: mouse anti-immunoglobulin G (IgG) (H+L) (115–035–003, Jackson ImmunoResearch, PA, USA) and rabbit anti-IgG (H+L) (111–035–003, Jackson ImmunoResearch). Sal (C14H20O7, CAS#: 10338–51–9, purity > 98%) and mitochondrial division inhibitor 1 (Mdivi-1) (CAS#: 338967-87-6, purity > 98%) were all purchased from MedChem Express (Monmouth Junction, NJ, USA). For the *in vitro* studies, Sal was dissolved in dimethyl sulfoxide (DMSO) and diluted with neuronal medium to final concentrations of 25, 50, 100, and 200 *μ*mol/mL. For the *in vivo* studies, Sal was dissolved in normal saline given intraperitoneally. The dose and treatment time of Sal both *in vivo* and *in vitro* were determined according to the relevant literature [31, 40].

### 2.3. Primary Spinal Neuron Culture

Embryonic (E16–E18) Sprague–Dawley (SD) rats were used to extract primary neurons according to established protocols [41]. Neurons, at a density of 5 × 10^4^ cells/mL and 1 × 10^6^ cells/mL, were seeded on 24-well and 6-well poly-D-lysine-coated plates (Corning Inc., Corning, NY, USA), respectively. After 4 h, the plates were gently tapped to remove nonneuronal cells that were not firmly attached to the plates and the medium was replaced with serum-free 96% neurobasal medium containing B27 (2%, *w*/*v*; Thermo Fisher Scientific, Waltham, MA, USA), glutamine (0.5 mM; Thermo Fisher Scientific), penicillin (100 IU/mL), and streptomycin (100 mg/mL; Thermo Fisher Scientific). The growth of neurons was observed under an inverted microscope. One half of the medium was changed every other day. When the cells were cultured for about 7 days, the purity of the obtained neurons was identified using anti-microtubule-associated protein 2 (MAP2) (1 : 500, rabbit IgG; Abcam) and neuronal nuclei antigen (1 : 800, mouse IgG; Abcam) under a fluorescence inversion microscope (AxioVert A1 and Imager A2; Carl Zeiss, Jena, Germany).

### 2.4. Nissl Staining

Mice were anesthetized with a lethal dose of chloral hydrate at 12 h after reperfusion and then perfused with normal saline and ice-cold paraformaldehyde (4%, *w*/*v*). L1–L5 segments of the spinal cord were then removed. After paraformaldehyde fixation and sucrose gradient dehydration, spinal cord tissues were embedded in optimum cutting temperature compound (Sakura, CA, USA) and cut into serial transverse sections to a thickness of 5 *μ*m. Spinal cord sections of each group were subjected to Nissl staining with Cresyl Violet (FD NeuroTechnologies, Columbia, MD, USA). Briefly, sections were washed with distilled water and stained for 10 min in a Cresyl Violet solution followed by differentiating with 95% ethanol, washing with xylene, and fixing with neutral balsam. Gray-matter damage was assessed by counting the number of normal motor neurons in the ventral part of the gray matter [25]. Cells that contained Nissl substance in the cytoplasm, loose chromatin, and prominent nucleoli were considered normal motor neurons. The number of surviving intact neurons per mm length of the spinal cord was counted in every fifth section (three slices per animal) at high magnification under a light microscope [42]. The results were expressed as the average number of cells within each frame per section. To reduce counting bias, cell counting was performed by two independent investigators blinded to treatment history.

### 2.5. Functional Locomotor Scores

The Basso mouse scale, which has a scale ranging from 0 (the poorest outcome) to 9 (the best outcome), was conducted at 6 and 12 h and at 1, 3, 7, and 14 days after reperfusion [43]. Nine points indicated the motor function of normal mice, while 0 points represented complete paralysis and 1–8 points represent varying degrees of neurological deficits. The inclined plate test [44, 45] was also carried out to access the motor function of mice. Briefly, the animals were placed on an inclined plane which could be adjusted to provide a slope of varying grade, and then, the maximum slope of the plane at which the animal could maintain its position for more than 5 s without falling was assessed. Both assessments were performed independently by two trained assessors who were blinded to the treatment history at a fixed time on each testing day. The average of the results was taken for statistical analysis.

### 2.6. Footprint Analysis

Gait and motor coordination were assessed 14 days after surgery. The front and rear paws were coated with dyes of different colors. Then, the mice were placed in a narrow passage padded with a white paper and encouraged to walk in a straight line. The obtained patterns were photographed with digital cameras and a representative image was used to assess coordination.

### 2.7. Oxygen-Glucose Deprivation/Reperfusion (OGD/R) Model

The OGD/R model was established as previously described [46, 47]. After washing with Dulbecco's Modified Eagle Medium (DMEM) without glucose (Thermo Fisher Scientific) [46], primary neurons were cultured in sugar-free medium and then placed in a modular incubator chamber that was flushed with 2 L/min of a 95% N_2_/5% CO_2_ gas mixture for 15 min at room temperature to remove oxygen. The chamber was then sealed and placed in a 37°C incubator as described previously [46]. Thirty minutes after OGD, neurons were placed in normal incubator and the sugar-free medium was replaced with normal medium for 12 h for further analysis.

### 2.8. Cell Viability Assay

Cell viability was assessed using the Cell Counting Kit-8 (CCK-8) assay (Dojindo, Kumamoto, Japan). Primary neurons were seeded in 96-well plates at a density of 1.5 × 10^4^ cells/mL and pretreated with different concentrations of Sal for 12 h, followed by treatment with OGD/R for 12 h. CCK-8 solution (10 *μ*L) was added to each well and incubated for 4 h in a humidified 5% CO_2_ atmosphere. The amount of orange formazan staining was calculated by measuring the absorbance at 450 nm using a microplate reader (ELx800; BioTek, Winooski, VT, USA). The average of six replicates for each group was calculated and used for analysis.

### 2.9. TUNEL Assay

Spinal cord sections acquired from mice and primary spinal cord neurons were used to implement the TUNEL protocol. Firstly, spinal cord sections were incubated with anti-NeuN overnight at 4°C after fixation, permeabilization, and blocking sections were incubated with Alexa Fluor 488-conjugated goat anti-mouse IgG antibodies for 2 h at room temperature. TUNEL assay was then performed to identify DNA fragmentation. Briefly, after washing with phosphate-buffered saline (PBS) three times, neurons and frozen spinal cord sections were stained with TUNEL reaction solution containing terminal deoxynucleotidyl transferase (Promega, Madison, WI, USA) for 1 h at 37°C according to the manufacturer's protocol [48]. After labeling, cell nuclei were counterstained with DAPI. The images were observed and photographed under a fluorescence microscope (AxioVert A1 and Imager A2). Nuclei that incorporated green fluorescence were determined to be apoptotic.

### 2.10. Measurement of Mitochondrial Membrane Potential (ΔΨ*m*)

ΔΨ*m* was measured using a commercial assay kit (Beyotime Biotechnology, Shanghai, China). In brief, neurons were incubated with JC-1 staining solution for 20 min at 37°C, washed with JC-1 staining buffer, and immersed in neuronal medium. Images of positively stained cells were taken using a fluorescence microscope. Normal mitochondria produced red fluorescence, and depolarized or inactive mitochondria produced green fluorescence. ΔΨ*m* was calculated as the ratio of red to green fluorescence.

### 2.11. Determination of ROS

A ROS detection kit was purchased from Beyotime Biotechnology. Briefly, primary neurons cultured in the 24-well plates were incubated with DCFH-DA in the culture chamber for 20 min in the dark, then washed three times with serum-free culture medium. ROS production was measured by DCF fluorescence at an excitation wavelength of 488 nm and an emission wavelength of 519 nm using a fluorescence microscope. The individual value of fluorescence intensity was normalized against that measured in untreated controls.

### 2.12. Evaluation of Oxidative Stress

The commercial kits for measurement of superoxide dismutase (SOD) activity and malondialdehyde (MDA), oxidized glutathione (GSSG), and reduced glutathione (GSH) concentrations in spinal cord tissues were purchased from Beyotime Biotechnology. Fresh spinal cord tissue was collected and ground to 100 g/L homogenates in a homogenizer with PBS. The homogenates were then centrifuged for 15 min at 4°C and incubated in radioimmunoprecipitation assay lysis buffer to determine the total protein content. Finally, SOD activity and MDA and GSSG/GSH concentrations in samples were measured and analyzed according to the manufacturer's protocol.

### 2.13. mt-Keima Lentivirus Transfection and Puncta Quantification

The mt-Keima lentivirus was purchased from Hanheng Biology Co., Ltd. (Shanghai, China), and titers were determined (1 × 10^8^). Primary spinal cord neurons were prepared and were seeded on confocal dishes for 4 days and then transfected with the mt-Keima virus according to the manufacturer's protocol. After 3–4 days, green fluorescence was observed in neurons, which indicated successful transfection, with green puncta representing normal mitochondria. After treatment with or without Sal, neurons were subjected to OGD/R. The images were taken using a Confocal Imaging System (Zeiss, Oberkochen, Germany, LSM 510); when the mitochondria fused with lysosomes, the puncta become red.

### 2.14. Transmission Electron Microscopy

As described in Animals and Experimental Protocol, after 12 h of I/R, the mice were perfused with precooled PBS, 4% paraformaldehyde, and 0.25% glutaraldehyde; the spinal cord tissue was removed. The tissue was fixed in a PBS mixture of 2% paraformaldehyde and 2.5% glutaraldehyde overnight at 4°C. The ischemic-reperfusion area was cut into 50 *μ*m cubes, fixed with osmium tetroxide for 1 h, dehydrated with a gradient of ethanol, and placed in an epoxy resin. After treatment for 24 h at 80°C, the specimen was sliced to a thickness of 100 nm and stained with uranyl acetic acid and lead citrate. Finally, images were photographed using a transmission electron microscope (Tecnai G2 Spirit Bio TWIN, FEI, USA).

### 2.15. Immunofluorescence Staining

Neurons or spinal cord sections of mice were fixed with polyformaldehyde (4%, *w*/*v*), permeabilized with 0.05% Triton X-100 for 15 min, blocked with 5% bovine albumin for 1 h, and incubated with primary antibodies overnight at 4°C. The neurons or tissue sections were treated then with Alexa Fluor 488- and Alexa Flour 594-conjugated goat secondary antibodies (1 : 200; Jackson ImmunoResearch) for 1 h at room temperature. Finally, DAPI (Thermo Fisher Science) was added to stain the nuclei after washing three times with PBS. Immunoreactivity was observed and photographed using an epifluorescence (AxioVertA1 and ImagerA2) or a confocal fluorescence microscope (LSM510; Carl Zeiss). At least four different samples were analyzed for each group, and each sample was randomly acquired in at least three visual fields. The staining intensities were measured by observers blinded to the experimental groups using ImageJ software (National Institutes of Health, Bethesda, MD, USA).

### 2.16. Western Blot

Whole-protein extraction kit and mitochondrial/cytoplasmic protein extraction kits (KeyGen Biotechnology, Shanghai, China) were used to extract protein according to the manufacturer's protocol. Protein was electrophoresed on an SDS-PAGE gel and transferred to PVDF membranes (SEQ00010; EMD Millipore) and blocked with bovine serum albumin (5%, *v*/*v*) followed by incubating overnight at 4°C with primary antibodies and then incubated for 2 h at room temperature with the secondary antibody. The membrane was visualized using chemiluminescent reagents (Pierce Biotechnology) and quantified using ImageJ software (National Institutes of Health).

### 2.17. Statistical Analysis

The data are presented as the mean values ± standard error mean (SEM) of at least three independent experiments. One-way ANOVA followed by Tukey's HSD post hoc test was used to measure differences between mean values of the different treated groups; *p* < 0.05 was considered significant. The values were analyzed using GraphPad Prism, version 7.0 (GraphPad Software, San Diego, CA, USA).

## 3. Results

### 3.1. Sal Promoted the Recovery of Motor Function in Mice and Reduced the Loss of Motor Neurons in the Anterior Horn of Spinal Cord after SCIRI

A schematic diagram of the experimental design is shown in Figure 1(a). The BMS score and inclined plate test were used to evaluate the effect of Sal on the recovery of motor function following SCIRI. The BMS score demonstrated that mice in the treatment group exhibited better performance than those in the SCIRI group at 1, 3, 7, and 14 days after SCIRI (Figure 1(b)). The results of the inclined plate test also showed that the maximum slope of the Sal treatment group was higher than that of the SCIRI group and both scores of the Sal high-dose group were higher than those of the low-dose group (Figure 1(c)). We also performed behavioral tests on mice 7 days after SCIRI. Specifically, the walking gait was evaluated by manually analyzing footprints (Figure 1(d)). After SCIRI, the coordination of the hind paw movements of all animals decreased significantly. Compared with the animals in the SCIRI group, the animals in the Sal treatment group showed significant recoveries of gait and motor coordination; and the improvement in Sal high-dose group was more pronounced. The results of the footprint experiment also showed that the rear limb movement function of the mice in the Sal group was significantly improved compared with that of the mice in the untreated group. In addition, the number of spinal motor neurons was measured by Nissl staining (Figure 1(e)). Results revealed that the number of Nissl bodies representing living motor neurons in the anterior horn of the spinal cord increased and the structure of Nissl bodies was clearer after Sal treatment 3 days after SCIRI compared with the non-Sal treatment. In conclusion, our results show that Sal was beneficial for the recovery of motor function in mice and the survival of neurons after SCIRI.

### 3.2. Sal Inhibited Intrinsic Apoptosis of Spinal Cord Neurons after SCIRI *In Vivo*

**Figure 2:**
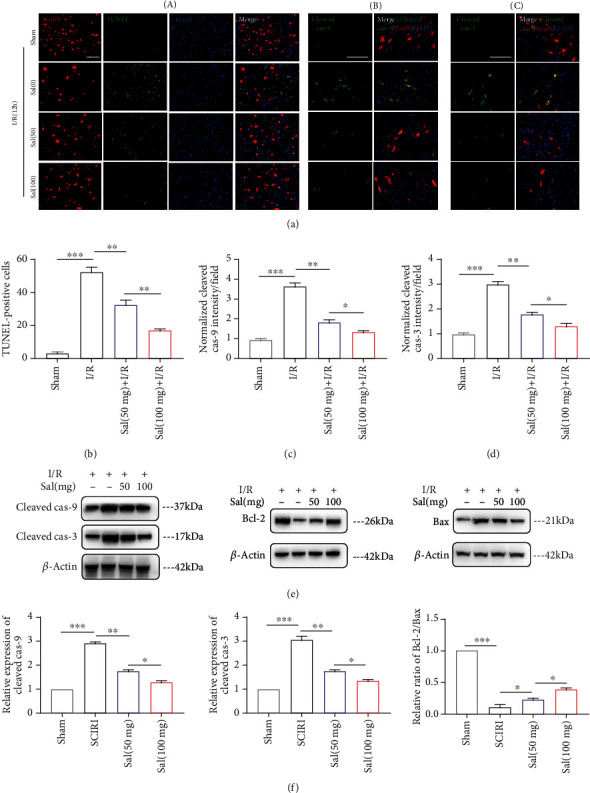
Sal inhibited intrinsic apoptosis of spinal cord neurons after SCIRI *in vivo*.(a, A) Representative images of NeuN, TUNEL, and DAPI costaining of spinal cord sections of mice that were subjected to sham operation or SCIRI for 12 h. Scale bar, 100 *μ*m. (a, B and C) Immunostaining of activated caspase-9 (B; in green) and activated caspase-3 (C; in green) in the spinal cords of mice. Representative images were colabeled with NeuN (in red) and DAPI. Scale bar, 100 *μ*m. Quantification of (b) TUNEL-positive cells and (c, d) cleaved caspase-3/caspase-9 intensity normalized to their respective levels in sham-operated mice. (e) Representative western blots of cleaved caspase-3/caspase-9, Bcl-2, and Bax expression in the spinal cords of mice that were subjected to sham operation or SCIRI for 12 h. (f) Quantification of activated caspase-3/caspase-9 and the ratio of Bcl-2 to Bax normalized to the levels in sham-operated mice on the basis of the western blots previously described. *β*-Actin was used as loading control. All values in this figure were presented as mean ± SEM (*n* = 4); ∗*p* < 0.05, ∗∗*p* < 0.01, and ∗∗∗*p* < 0.001.

Emerging evidence suggests that apoptosis is the basis for neuronal loss [7]. Therefore, we further used the TUNEL protocol and the neuronal cell marker protein (NeuN) to evaluate the apoptosis of neurons (Figures 2(a), A and 2(b)). A large number of TUNEL-positive neurons were detected after SCIRI, while this number was significantly reduced in spinal cord sections of Sal-pretreated mice, with the reduction being more pronounced in the high-dose Sal-treated group. The expression levels of caspases are indicators of the intrinsic apoptotic phenotype [49]. Cleaved caspase-9, an important initiator of apoptosis, has been implicated in nervous system injury [50, 51]. Caspase-3 is an executioner of apoptosis via its function in cleaving several essential downstream substrates [51, 52]. Immunostaining results (Figures 2(a), B and C, 2(c), and 2(d)) demonstrated that cleaved caspase-3/caspase-9 expression was mainly observed in neurons in response to I/R, and the fluorescence intensity of cleaved caspase-3/caspase-9 in neurons was lower in the Sal treatment group than in the vehicle treatment group after SCIRI. Results of western blotting (Figures 2(e) and 2(f)) demonstrated that the levels of cleaved caspase-3/caspase-9 in the spinal cord were considerably decreased in Sal-treated mice. The Bcl-2/Bax ratio is known to be inversely related to neuronal apoptosis [53], demonstrating a significant increase in the Sal treatment group after I/R compared with vehicle mice. Collectively, these results suggest a protective function of Sal against spinal cord neuronal apoptosis in response to I/R.

### 3.3. Sal Reduced the Apoptosis of Primary Neurons and Promoted Neurons Survival after OGD/R *In Vitro*

**Figure 3:**
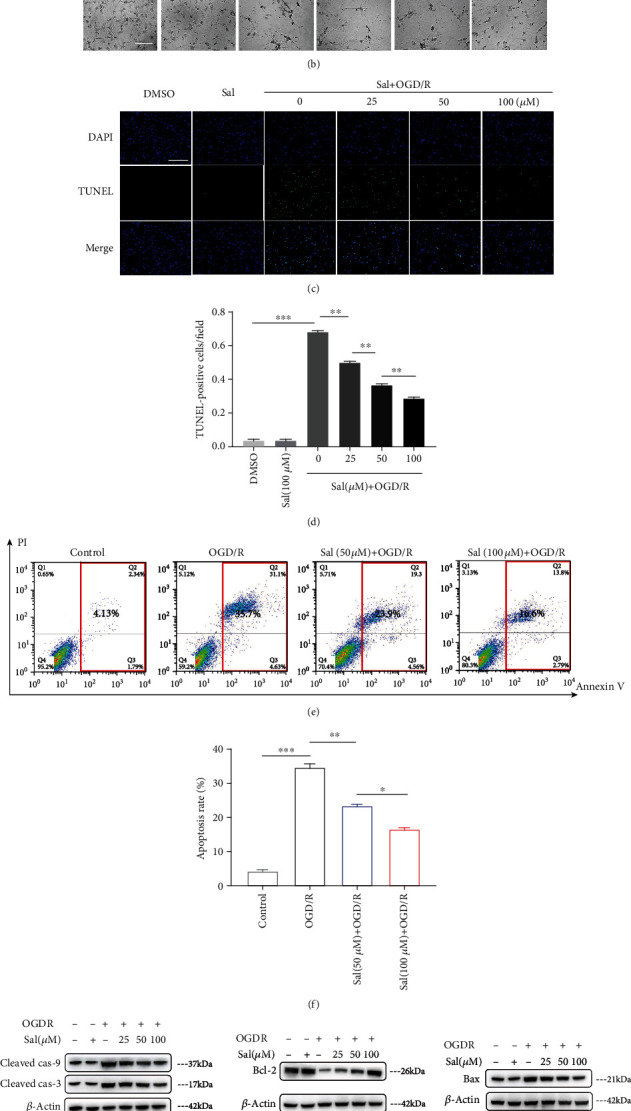
Sal reduced the apoptosis of primary neurons and promoted neuronal survival after OGD/R *in vitro*. (a) The CCK-8 kit was used to detect the viability of neurons that were treated with different concentrations of Sal. (b) Brightfield images showing morphologic changes of primary neurons in control and OGD/R-treated samples in the presence or absence of different concentrations of Sal before OGD/R for 12 h. Scale bar, 100 *μ*m. (c) Representative images of TUNEL and DAPI costaining of spinal cord neurons that were subjected to control or OGD/R for 12 h. Scale bar, 100 *μ*m. (d) Quantification of apoptotic (TUNEL+) cells was performed (*n* = 6). (e) Examples of scatter plots for neurons that were subjected to control or OGD/R for 12 h by PI/Annexin V double labeling. (f) Quantification of apoptotic cells in (c). (g) Representative western blots of cleaved caspase-3/caspase-9, Bcl-2, and Bax expression in neurons that were subjected to control or OGD/R for 12 h. (h) Quantification of activated caspase-3/caspase-9 expression and the ratio of Bcl-2 to Bax normalized to the level in neurons that were treated with DMSO on the basis of the western blots previously described. *β*-Actin was used as loading control. All values in this figure were presented as mean ± SEM (*n* = 4); ∗*p* < 0.05, ∗∗*p* < 0.01, and ∗∗∗*p* < 0.001.

In order to further verify the protective effect of Sal on spinal cord neurons after SCIRI, we isolated neurons from fetal mouse spinal cord for culture *in vitro*; the purity of spinal cord neurons was confirmed by immunostaining MAP2 (Supplementary material 1), a mature neuron marker protein, after 1 week of culture. OGD/R was performed to simulate SCIRI *in vivo*, whereby primary neurons were treated with or without different concentrations of Sal for 12 h prior to OGD/R. The CCK-8 assay, used to determine the cell viability, showed that Sal did not have an obvious toxic effect on neurons cultured *in vitro* (Figure 3(a)). At the same time, in a range of concentrations, the survival rate of OGD/R-treated neurons was positively correlated with the concentration of Sal. After exposure to OGD/R for 12 h, neuronal loss and breakdown of neurites were clearly observed (Figure 3(b)); however, in the Sal treatment group, the number and morphology of neurons were significantly improved, and the improvement was more pronounced with increasing Sal concentrations. Similarly, TUNEL assay *in vitro* showed that Sal significantly reduced the neuronal apoptosis in a dose-dependent manner after OGD/R (Figures 3(c) and 3(d)). In addition, flow cytometry was performed to detect apoptosis. Exposure to OGD/R significantly increased neuronal apoptosis without Sal pretreatment as measured by Annexin V-FITC/PI double staining. However, this response was markedly attenuated when cells were pretreated with Sal for 12 h prior to OGD/R (Figures 3(e) and 3(f)). Western blotting also demonstrated that the expression of intrinsic apoptosis-related protein cleaved caspase-3/caspase-9 and Bax in primary neurons in response to OGD/R treatment was significantly increased, while the expression of apoptosis-inhibiting protein Bcl-2 was downregulated. However, this phenomenon was significantly reversed after pretreatment with Sal, and the reversal was more evident as the concentration of Sal was increased (Figures 3(g) and 3(h)). Overall, *in vitro* results were consistent with *in vivo* data, which indicated that Sal was beneficial for the reduction of neuronal apoptosis after OGD/R and promoted the survival of spinal cord neurons.

### 3.4. Sal Inhibited Oxidative Stress and Decreased the Damage of ROS to Mitochondria

**Figure 4:**
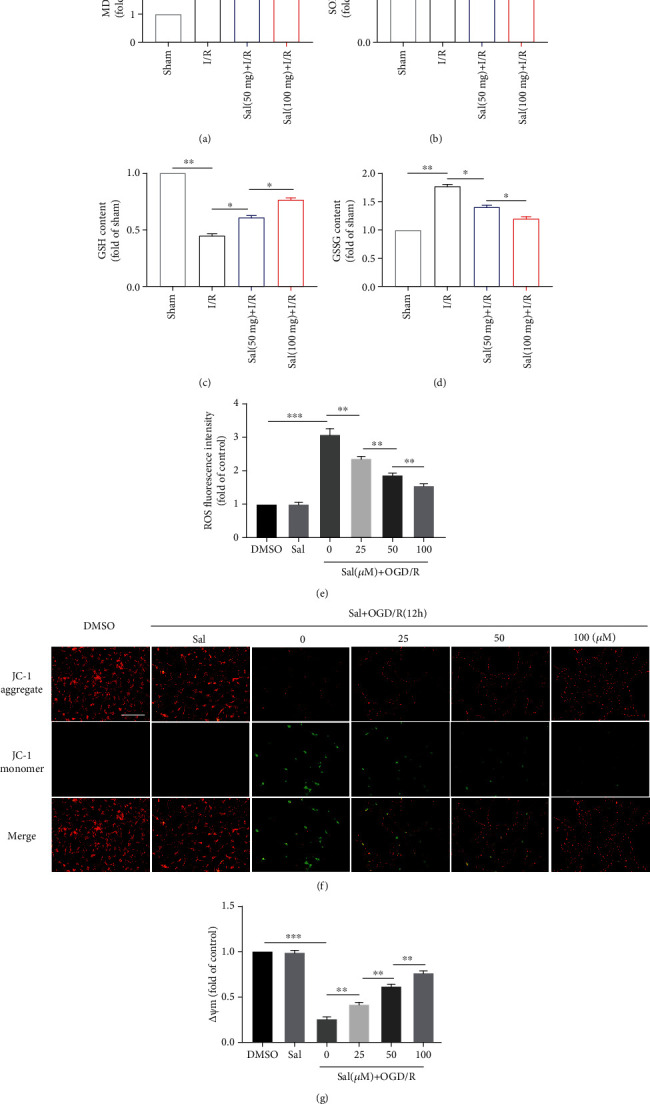
Sal inhibited oxidative stress and lessened the damage of reactive oxygen species to mitochondria. (a–d) SOD activity and MDA, GSSG, and GSH concentrations in the spinal cord were determined according to the respective assay kit results. (e) The levels of intracellular ROS in neurons were determined by a ROS assay kit. (f) Representative images of immunofluorescence of JC-1, indicative of ΔΨ*m*. Scale bar, 100 *μ*m. (g) Quantitative analysis of the change of the mitochondrial green/red fluorescence ratio. All values in this figure were presented as mean ± SEM (*n* = 4); ∗*p* < 0.05, ∗∗*p* < 0.01, and ∗∗∗*p* < 0.001.

Oxidative stress plays an important role in SCIRI, and ROS mediates a series of injury responses. Sal has a good antioxidant capacity, and in order to determine the local oxidative/antioxidative levels in response to Sal, we detected SOD activity and MDA, GSH, and GSSG concentrations in spinal cord tissue. Spinal cord I/R was associated with a significant elevation in MDA and GSSG concentrations with concomitant reduction in SOD activity and GSH concentration. Following SCIRI, SOD activity and GSH concentration in the Sal-treated group were significantly higher than those in the SCIRI group, but MDA and GSSG concentrations were decreased significantly compared with those in the SCIRI group. Again, the change was more pronounced in the high-dose Sal treatment group (Figures 4(a)–4(d)). After OGD/R, ROS levels in spinal cord neurons cultured *in vitro* were also detected. As expected, ROS levels increased significantly after OGD/R, while after Sal pretreatment, ROS levels decreased with the increase in Sal concentration (Figure 4(e)). After SCIRI, the production of a large amount of ROS will lead to oxidative stress, and mitochondria are one of the main targets. Mitochondrial damage was initially characterized by a decrease in membrane potential. Hence, JC-1 fluorescent probes were used to indicate changes in ΔΨ*m*; when the ΔΨ*m* is normal, JC-1 will accumulate in the mitochondrial matrix and emit red fluorescence, whereas when the mitochondrial membrane potential is reduced or collapsed, JC-1 is not able to accumulate and emits a green fluorescence. It was found that normal neurons emitted red fluorescence and green fluorescence was rarely observed; however, 12 h after OGD/R, the intensity of red fluorescence was greatly reduced and green fluorescence was significantly enhanced. Notably, Sal restored the loss of ΔΨ*m* after OGD/R, which was indicated by the reversal of fluorescence intensity, and within a certain range, the higher the concentration of Sal, the higher the ΔΨ*m* (Figures 4(f) and 4(g)).

### 3.5. Sal Enhanced Mitophagy after SCIRI

**Figure 5:**
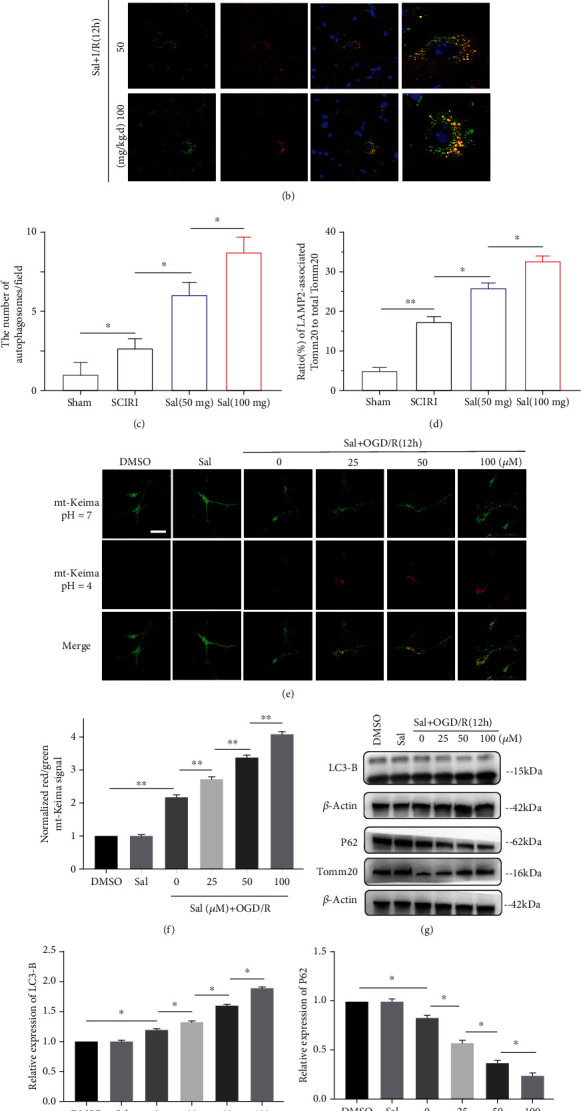
Sal enhanced mitophagy after SCIRI. (a) Electron micrographs demonstrating neuron mitophagy structure in Sal-treated SCIRI mice. Typical mitophagy structure (white arrow) was observed in neurons. The high magnifications of lower box revealed a typical structure of (A) normal mitochondria, (B) swollen mitochondria, (C) autolysosomes, and (D) mitophagy from the four groups of mice mentioned above. Scale bar, 0.5 *μ*m. (b) Immunohistochemical colocalization of Tomm20 (mitochondrial marker, green) and LAMP2 (lysosomal marker, red) in the spinal cords from the four groups of mice mentioned above at 12 h after SCIRI. The inset images represent higher magnification of the boxed area in the corresponding merged images. Scale bar, 100 *μ*m. (c) Quantification of the ratio of mitochondria engulfed by autophagosomes to total mitochondria in each field. (d) Statistical analysis of the ratio of LAMP2-associated Tomm20 to total Tomm20. (e) Representative images of mt-Keima to detect normal mitochondria (in green) and mitochondria in autophagosomes (in red), in neurons. Neurons were treated with DMSO, different concentrations of Sal, with or without OGD/R for 12 h. Scale bar, 100 *μ*m. (f) Quantification of the ratio of red to green dots in neurons. Representative (g) western blots and (h–j) quantitative graphs demonstrated the expression of LC3-B, P62, and Tomm20 in whole homogenates normalized to the level of *β*-actin. All values are presented as mean ± SEM (*n* = 4); ∗*p* < 0.05, ∗∗*p* < 0.01, and ∗∗∗*p* < 0.001.

Autophagy is a ubiquitous and unique life phenomenon of eukaryotic cells. Mitophagy is a special type of autophagy, which can specifically engulf damaged mitochondria to maintain mitochondrial homeostasis [54]. Emerging evidence suggested a neuroprotective role of mitophagy in cerebral I/R [21, 23], and enhancing mitophagy attenuated apoptosis after SCIRI [24]. Therefore, mitophagy may play a pivotal role in neuronal survival during SCIRI. We further investigated the effect of Sal on mitophagy after SCIRI. Transmission electron microscopy (Figure 5(a)) showed that the mitochondria of normal neurons were short rod-shaped or round, dark, and clearly structured (Figure 5(a), A). However, 12 h after SCIRI, the mitochondria were swollen and round, the matrix was weak or even bright, and the ridge structure disappeared (Figure 5(a), B). Meanwhile, a small number of cracked mitochondria were found to be surrounded by double-layer vesicles (Figure 5(a), C and D), indicative of an autophagosome, which increased after Sal treatment (Figures 5(a) and 5(c)). After the damaged mitochondria are encased in autophagosomes, the next step is to fuse with the lysosomes to degrade the mitochondria. Hence, the colocalization of LAMP2, a lysosomal surface marker protein and mitochondrial outer membrane protein TOMM20, was observed. The results (Figures 5(b) and 5(d)) showed that the colocalization was slightly increased in mice after SCIRI while mitochondria and lysosomes were independent of each other and rarely colocalized under normal conditions. However, more colocalization was observed in the spinal cord sections of mice pretreated with Sal, and colocalization was further increased in the high-dose Sal treatment group. *In vitro*, transfection of mt-Keima lentivirus into spinal cord neurons represented mitophagy. mt-Keima-labeled mitochondria emit green fluorescence under physiological conditions (pH = 7), but when mitophagy occurs, mitochondria are engulfed by lysosomes in an acidic environment (pH = 4) and emit red fluorescence. After OGD/R for 12 h, we found red puncta in spinal cord neurons, and Sal pretreatment prior to OGD/R further increases the number of red puncta in a dose-dependent manner (Figures 5(e) and 5(f)). Similar to immunofluorescent assay results, western blot showed that Sal increased autophagy flux, as evidenced by an increase in LC3-B expression and a decrease in p62 and Tomm20 expression (Figures 5(g)–5(i)). Various experiments both *in vivo* and *in vitro* have shown that Sal can promote the occurrence of neuronal mitophagy after SCIRI.

### 3.6. Sal Promoted Mitophagy via Modulating the PINK1-Parkin Signaling Pathway

**Figure 6:**
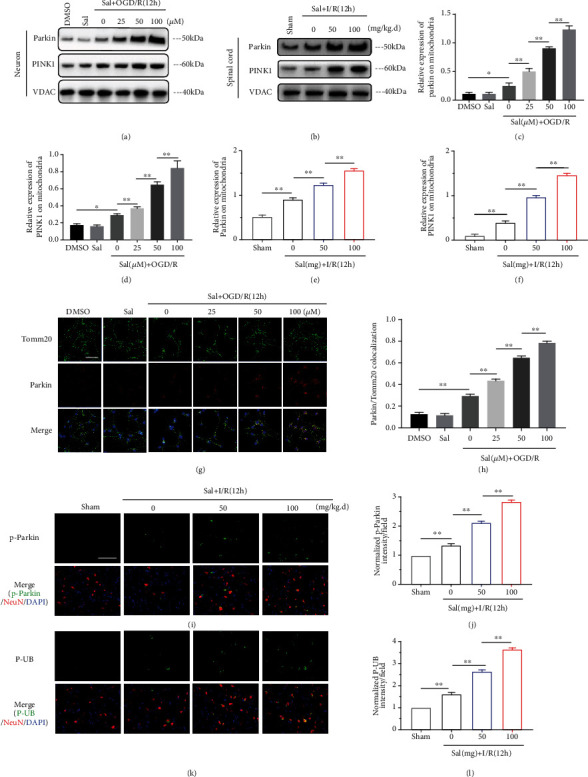
Sal promoted mitophagy via modulating the PINK1-Parkin signaling pathway. Representative western blots of the expression of mitochondrial proteins (Parkin and PINK1) in the (a) neurons and (b) spinal cords that were subjected to OGD/R or I/R for 12 h, respectively. (c–f) Quantification of Parkin and PINK1 protein expression normalized to the level of VDAC. Representative (g) confocal images and (h) statistical analysis of the colocalization of Parkin with mitochondrial neurons. The neurons were treated with DMSO and different concentrations of Sal, with or without OGD/R for 12 h. Scale bar, 100 *μ*m. (i, k) Representative images of immunofluorescent labeling of p-Parkin (i), p-ubiquitin (k, in green), NeuN (in red), and DAPI (in blue) in the spinal cords of mice that underwent sham operation or ischemia-reperfusion injury for 12 h. Scale bars, 200 *μ*m. Statistical analysis based on the expression of (j) p-Parkin and (l) p-ubiquitin from mice in the sham group. All values are presented as mean ± SEM (*n* = 4); ∗*p* < 0.05, ∗∗*p* < 0.01, and ∗∗∗*p* < 0.001.

To explore the mechanism of mitophagy activated by Sal, we measured mitochondrial Parkin and PINK1 after SCIRI, which are key mediators for mitophagy. After mitochondrial injury, PINK1, which is usually degraded in the cytoplasm, accumulates on the outer membrane of the damaged mitochondria, phosphorylating Parkin protein in the cytoplasm and promoting its transfer to the mitochondria. Parkin is an E3 ubiquitin ligase, which can promote the ubiquitination of mitochondrial membrane proteins and which then binds to autophagosome protein LC3II to induce mitophagy [55–57]. Western blot and immunofluorescence results showed that Sal could significantly strengthen I/R-induced increases in PINK1 and Parkin expression in mitochondria (Figures 6(a)–6(h)). In the Sal treatment group, we also found that, following Parkin translocation to the mitochondria, the expression levels of the effector proteins, p-Parkin and P-UB [56, 58], were significantly increased in neurons (Figures 6(i)–6(l)). These findings support the role of PINK1/Parkin signaling in Sal-induced mitophagy.

### 3.7. Mdivi-1, a Specific Mitophagy Inhibitor, Partially Reversed the Protective Effect of Sal on Spinal Cord Neurons

**Figure 7:**
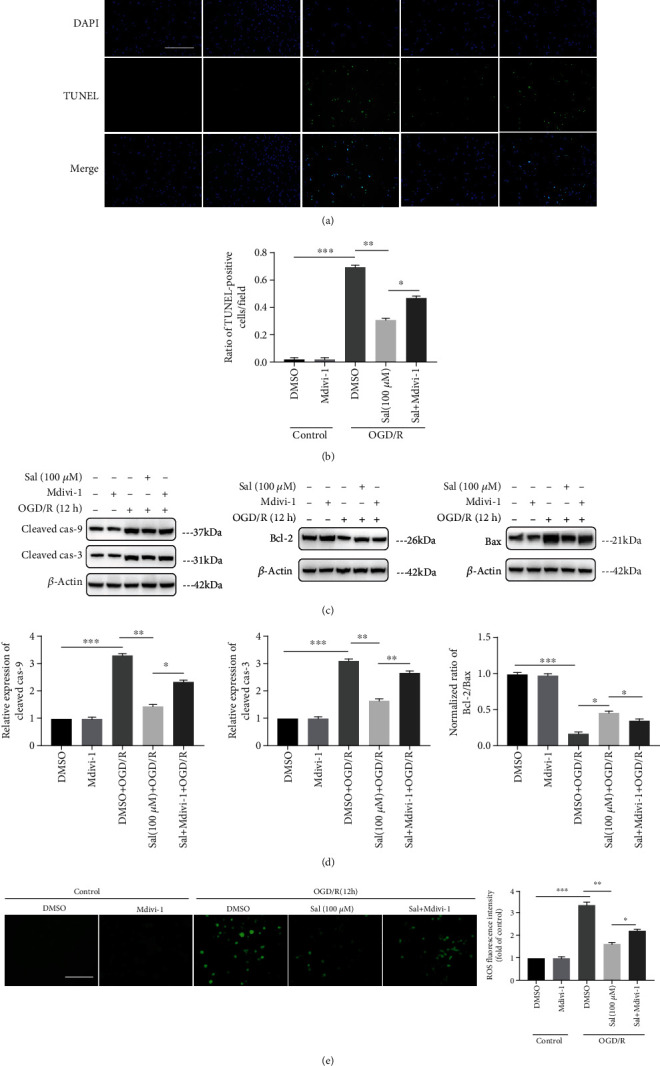
Mdivi-1, a specific mitophagy inhibitor, partially reversed the protective effect of Sal on spinal cord neurons. (a) Representative images of TUNEL and DAPI costaining of spinal cord neurons that were subjected to control or OGD/R for 12 h. Scale bar, 100 *μ*m. (b) Quantification of apoptotic (TUNEL+) cells was performed. (c) Representative western blots of cleaved caspase-3/caspase-9, Bcl-2, and Bax expression in neurons that were subjected to control or OGD/R for 12 h. (d) Quantification of activated caspase-3/caspase-9 expression and the ratio of Bcl-2 to Bax normalized to the level of neurons in the control group that were pretreated with DMSO. GAPDH was used as a loading control. (e) Representative images show ROS production in spinal cord neurons and quantification of ROS intensity normalized to the level in control neurons. Scale bar, 100 *μ*m. Values are expressed as the ratio of fluorescence intensity relative to the control. All values are expressed as mean ± SEM (*n* = 4); ∗*p* < 0.05, ∗∗*p* < 0.01, and ∗∗∗*p* < 0.001.

Mitochondria-dependent apoptosis is associated with release of cytochrome *C* into the cytoplasm following SCIRI [59]. To further verify whether Sal played a neuroprotective role by promoting the occurrence of mitophagy, Mdivi-1 [60, 61], a specific inhibitor of mitophagy by preventing mitochondrial division, was used to treat neurons to inhibit neuronal mitophagy [62]. Apoptosis of spinal cord neurons in vitro was also detected by TUNEL protocol. Similarly, Sal pretreatment significantly reduced the number of TUNEL-positive cells after OGD/R, but this effect was partially abrogated after Mdivi-1 treatment (Figures 7(a) and 7(b)). At the same time, western blot results showed that the expression of apoptosis-related protein Bax and cleaved caspase-3/caspase-9 was increased and that the expression of the apoptosis-inhibiting protein Bcl-2 was reduced (Figures 7(c) and 7(d)). Moreover, we also found that ROS levels increased after treatment with Mdivi-1 (Figure 7(e)), which may be associated with decreased clearance of damaged mitochondria after inhibition of mitophagy, resulting in increased release of ROS from damaged mitochondria [63]. Taken together, the results suggest that Sal's protective effect on neurons during SCIRI was at least partially due to increased mitophagy.

## 4. Discussion

I/R injury is the most common secondary injury of the spinal cord [64]. Approximately 11%–40% of patients undergoing spinal orthomorphia or excision of a thoracoabdominal aortic aneurysm will result in immediate or delayed paraplegia, which produce long-term morbidity and huge medical expenses for patients worldwide [65, 66]. In recent years, new or complementary medicinal products, in particular medicine food homology products, have shown potential for treatment applications worldwide due to their minimal side effects. In the present study, we demonstrated for the first time that Sal administration protected spinal cord neurons after SCIRI by reducing apoptosis. The main mechanisms involved suppressing oxidative stress and promoting mitophagy.

ROS play an important role in activating and regulating mitochondrial-mediated apoptosis [67]. Oxidative stress after ischemia-reperfusion injury produces a large number of ROS, causing oxidation/antioxidant system disorder in the body. Moreover, the accumulation of ROS leads to the opening of the mitochondrial permeability transition pore (MPTP) in the inner membrane [68], which decreased ΔΨ*m* and the release of cytochrome C into the cytoplasm to induce apoptosis cascade events [69]. Therefore, antioxidant stress is an important target in the treatment of spinal cord ischemia-reperfusion injury. Sal is a natural antioxidant that has shown potent antioxidant capacity in multiple systemic disease models [70–72], but its role in SCIRI is unclear. Here, *in vivo*, we found that Sal could significantly correct the imbalance of the oxidation/antioxidant system after SCIRI, while *in vitro* Sal reduced the production of ROS and restored the ROS-mediated ΔΨ*m* collapse in a dose-dependent manner. In summary, our results suggest that Sal protected the spinal cord from ischemia-reperfusion injury by reducing oxidative stress.

Damaged mitochondria release proapoptotic proteins to increase the activation of caspases and cell death [8]. Hence, in addition to controlling the source of mitochondrial damage, the removal of damaged mitochondria is also essential for cellular survival. The selective degradation of damaged mitochondria by autophagy is termed mitophagy, which is extremely crucial in maintaining mitochondrial homeostasis and has been implicated in I/R injury [21, 23, 73]. Emerging evidence has shown that Sal is closely related to the occurrence of mitophagy. In MPTP-induced Parkinson's disease models, Sal can enhance the mitochondrial expression of PINK1 and Parkin and confer neuroprotective effects [31]. Another study showed that upregulation of Parkin by Sal promoted the survival of nucleus pulposus cells through activation of mitophagy *in vitro* [74]. Therefore, we evaluated the occurrence of mitophagy in Sal after SCIRI. Consistent with previous reports, we found that the flux of mitophagy in the spinal cord of mice was significantly increased 12 h after SCIRI, which was further confirmed by transmission electron microscopy. The above results were further supported by immunofluorescence results that indicated an increase in the colocalization of mitochondria and lysosomes and in expression levels of the autophagy proteins LC3-B and P62. In addition, both *in vitro* and *in vivo*, we found that Sal could significantly improve mitophagy after SCIRI and the flux was increased with the increase in Sal concentration. Sal also increased the expression of PINK1 and Parkin after SCIRI and in particular Parkin expression in mitochondria, which suggested that Sal might promote mitophagy through the classical PINK1-Parkin signaling pathway. The expression of p-Parkin and P-UB, which are downstream proteins of the Parkin pathway, was found to be elevated in spinal cord neurons *in vivo*, which further confirmed our hypothesis. Overall, Sal promoted mitophagy after SCIRI by enhancing the PINK1-Parkin pathway.

The role of autophagy in SCIRI remains controversial. Some studies have shown that enhancing autophagy at the early stage of ischemia-reperfusion injury can reduce apoptosis and play a protective role [24, 25]. In contrast, others have reported that excessive autophagy is one of the main causes of cell death [22, 75]. In this study, we showed that mitophagy was beneficial in reducing apoptosis and promoting neuronal survival. More importantly, we showed that the protective effect associated with Sal treatment on neurons after OGD/R was partially abolished by the mitophagy inhibitor, Mdivi-1. This result suggested that the neuronal protective effect of Sal was, at least, partially mediated by mitophagy.

There were several limitations in the current study. For example, the effect of Sal on glial cells after SCIRI was not explored. In the early stage of SCIRI, glial cells respond quickly and act as a defense barrier against stimuli [76]. Inflammatory cells may participate in SCIRI by expressing inflammatory cytokines and inducing neuronal apoptosis and even death [77–79]. Sal has been reported to affect glial cell polarization and protect neurons in models of spinal cord injury and cerebral ischemia disease [33, 34, 80]. Hence, in addition to acting directly on neurons, Sal may also indirectly influence the fate of neurons through glial cells or other pathways that have yet to be elucidated. More comprehensive studies on the effects of Sal on cells other than neurons after SCIRI are necessary. Second, although we demonstrated that Sal increased the expression of PINK1 and Parkin in the mitochondria of neurons after SCIRI, the molecular pathways associated with Sal-induced PINK1 and Parkin expression should be further characterized.

**Figure 8:**
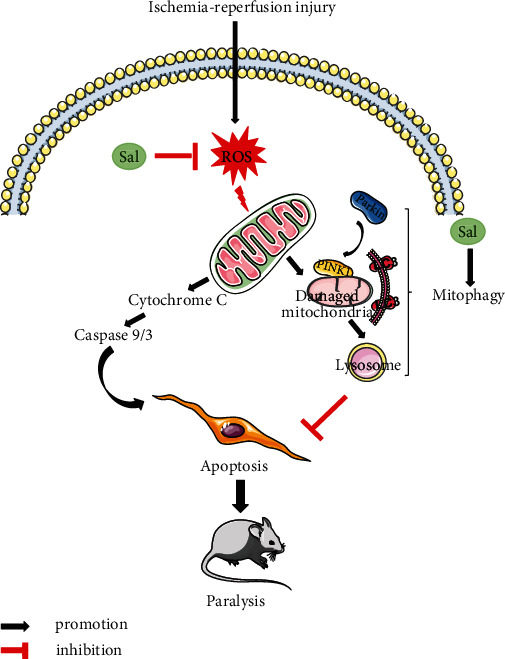
Sal ameliorates mitochondria-dependent neuronal apoptosis after spinal cord ischemia-reperfusion injury partially through inhibiting oxidative stress and promoting mitophagy. Oxidative stress occurs in spinal cord neurons after I/R or OGD/R, resulting in ROS that damage mitochondria, leading to changes in ΔΨ*m* and the release of large amounts of cytochrome C to induce caspase-mediated endogenous apoptosis. The loss of neurons will eventually lead to impaired neuronal function, such as paralysis. Multiple bioactive drugs including Sal can, on the one hand, reduce the level of damage to mitochondria by inhibiting oxidative stress and can, on the other hand, strengthen PINK1-Parkin pathway-mediated mitophagy in the early stage of spinal cord ischemia-reperfusion injury to accelerate the clearance of damaged mitochondria.

In conclusion, we have shown for the first time that Sal has a protective effect in a mouse model of SCIRI, which was verified by a significant functional recovery and a reduction of motor neuron loss in the anterior horn. The protective mechanism is associated with mitochondrial survival. The effects of Sal on mitochondria were combinatorial (Figure 8); on the one hand, Sal attenuated mitochondrial injury by eliminating ROS; and on the other hand, Sal promoted clearance of damaged mitochondria to maintain mitochondrial homeostasis through mitophagy. Finally, Sal was shown to reduce neuronal apoptosis induced by mitochondrial injury. Therefore, Sal treatment is a promising and effective therapeutic strategy for SCIRI.

## Data Availability

Data used to support the findings of this study are available from the corresponding author upon request.

## Abbreviations

SCIRI: Spinal cord ischemia-reperfusion injury
OGD/R: Oxygen-glucose deprivation/reperfusion
Sal: Salidroside
BMS: Basso mouse scale
ROS: Reactive oxygen species
MDA: Malondialdehyde
SOD: Superoxide dismutase
GSH: Reduced glutathione
GSSG: Oxidized glutathione
PBS: Phosphate-buffered saline
MAP2: Microtubule-associated protein 2
NeuN: Neuronal nuclei
PINK1: PTEN-induced kinase 1
VDAC: Anti-voltage-dependent anion channel.

## Supplementary Materials

Supplementary Materials Immunocytochemical identification of primary mouse spinal cord neurons. Neuronal dendrites and axons were identified by anti-MAP2 (green) and somata by NeuN (red) immunostaining. The nuclei of all cells were identified by DAPI (blue). Scale bars, 100 *μ*m. Neuronal cultures with cell purity greater than 90 percent were used in this study.

## References

[1] Svensson L. G., Crawford E. S., Hess K. R., Coselli J. S., Safi H. J. (1993). Experience with 1509 patients undergoing thoracoabdominal aortic operations. *Journal of vascular surgery*.

[2] Karadimas S. K., Laliberte A. M., Tetreault L. (2015). Riluzole blocks perioperative ischemia-reperfusion injury and enhances postdecompression outcomes in cervical spondylotic myelopathy. *Science translational medicine*.

[3] Li X. Q., Lv H. W., Tan W. F., Fang B., Wang H., Ma H. (2014). Role of the TLR4 pathway in blood-spinal cord barrier dysfunction during the bimodal stage after ischemia/reperfusion injury in rats. *Journal of Neuroinflammation*.

[4] Greenberg R. K., Lu Q., Roselli E. E. (2008). Contemporary analysis of descending thoracic and thoracoabdominal aneurysm repair: a comparison of endovascular and open techniques. *Circulation*.

[5] Wu Y., Satkunendrarajah K., Fehlings M. G. (2014). Riluzole improves outcome following ischemia-reperfusion injury to the spinal cord by preventing delayed paraplegia. *Neuroscience*.

[6] Foley L. S., Fullerton D. A., Bennett D. T. (2015). Spinal cord ischemia-reperfusion injury induces erythropoietin receptor expression. *The Annals of thoracic surgery*.

[7] Fricker M., Tolkovsky A. M., Borutaite V., Coleman M., Brown G. C. (2018). Neuronal cell death. *Physiological Reviews*.

[8] Anne Stetler R., Stetler R. A., Leak R. K., Gao Y., Chen J. (2012). The dynamics of the mitochondrial organelle as a potential therapeutic target. *Journal of Cerebral Blood Flow & Metabolism*.

[9] Niatsetskaya Z. V., Sosunov S. A., Matsiukevich D. (2012). The oxygen free radicals originating from mitochondrial complex I contribute to oxidative brain injury following hypoxia-ischemia in neonatal mice. *The Journal of neuroscience: the official journal of the Society for Neuroscience*.

[10] Sullivan P. G., Krishnamurthy S., Patel S. P., Pandya J. D., Rabchevsky A. G. (2007). Temporal characterization of mitochondrial bioenergetics after spinal cord injury. *Journal of Neurotrauma*.

[11] Fang S.-Y., Roan J.-N., Lee J.-S. (2019). Transplantation of viable mitochondria attenuates neurologic injury after spinal cord ischemia. *The Journal of Thoracic and Cardiovascular Surgery*.

[12] Kubli D. A., Gustafsson A. B. (2012). Mitochondria and mitophagy: the yin and yang of cell death control. *Circulation Research*.

[13] Halladin N. L. (2015). Oxidative and inflammatory biomarkers of ischemia and reperfusion injuries. *Danish Medical Journal*.

[14] Wang Y., Zhang S. X. L., Gozal D. (2010). Reactive oxygen species and the brain in sleep apnea. *Respiratory Physiology & Neurobiology*.

[15] Holzerova E., Prokisch H. (2015). Mitochondria: much ado about nothing? How dangerous is reactive oxygen species production?. *The International Journal of Biochemistry & Cell Biology*.

[16] Yang S., Li H., Tang L. (2015). Apelin-13 protects the heart against ischemia-reperfusion injury through the RISK-GSK-3*β*-mPTP pathway. *Archives of medical science : AMS*.

[17] Fu D., Liu H., Li S., Chen L., Yao J. (2017). Antioxidative and antiapoptotic effects of delta-opioid peptide [D-Ala^2^, D-Leu^5^] enkephalin on spinal cord ischemia-reperfusion injury in rabbits. *Frontiers in Neuroscience*.

[18] Topsakal C., Kilic N., Ozveren F. (2003). Effects of prostaglandin E1, melatonin, and oxytetracycline on lipid peroxidation, antioxidant defense system, paraoxonase (PON1) activities, and homocysteine levels in an animal model of spinal cord injury. *Spine*.

[19] Xie L., Yu S., Yang K., Li C., Liang Y. (2017). Hydrogen sulfide inhibits autophagic neuronal cell death by reducing oxidative stress in spinal cord ischemia reperfusion injury. *Oxidative Medicine and Cellular Longevity*.

[20] Matsui Y., Takagi H., Qu X. (2007). Distinct roles of autophagy in the heart during ischemia and reperfusion: roles of AMP-activated protein kinase and Beclin 1 in mediating autophagy. *Circulation Research*.

[21] Yuan Y., Zhang X., Zheng Y., Chen Z. (2015). Regulation of mitophagy in ischemic brain injury. *Neuroscience Bulletin*.

[22] Shen M., Lu J., Dai W. (2013). Ethyl pyruvate ameliorates hepatic ischemia-reperfusion injury by inhibiting intrinsic pathway of apoptosis and autophagy. *Mediators of Inflammation*.

[23] Zhang X., Yan H., Yuan Y. (2014). Cerebral ischemia-reperfusion-induced autophagy protects against neuronal injury by mitochondrial clearance. *Autophagy*.

[24] Li Q., Gao S., Kang Z. (2018). Rapamycin enhances mitophagy and attenuates apoptosis after spinal ischemia-reperfusion injury. *Frontiers in Neuroscience*.

[25] Liu K., Yan L., Jiang X. (2017). Acquired inhibition of microRNA-124 protects against spinal cord ischemia- reperfusion injury partially through a mitophagy-dependent pathway. *The Journal of Thoracic and Cardiovascular Surgery*.

[26] Mao G. X., Xing W. M., Wen X. L. (2015). Salidroside protects against premature senescence induced by ultraviolet B irradiation in human dermal fibroblasts. *International Journal of Cosmetic Science*.

[27] Zhang X. R., Fu X. J., Zhu D. S. (2016). Salidroside-regulated lipid metabolism with down-regulation of miR-370 in type 2 diabetic mice. *European Journal of Pharmacology*.

[28] Chiang H. M., Chen H. C., Wu C. S., Wu P. Y., Wen K. C. (2015). Rhodiola plants: chemistry and biological activity. *Journal of Food and Drug Analysis*.

[29] Cui J. L., Guo T. T., Ren Z. X., Zhang N. S., Wang M. L. (2015). Diversity and antioxidant activity of culturable endophytic fungi from alpine plants of *Rhodiola crenulata, R. angusta,* and *R. sachalinensis*. *PloS one*.

[30] Li T., Zhang W., Kang X. (2020). Salidroside protects dopaminergic neurons by regulating the mitochondrial MEF2D-ND6 pathway in the MPTP/MPP^+^ -induced model of Parkinson’s disease. *Journal of Neurochemistry*.

[31] Li R., Chen J. (2019). Salidroside protects dopaminergic neurons by enhancing PINK1/Parkin-mediated mitophagy. *Oxidative Medicine and Cellular Longevity*.

[32] Han T. (2013). Effects of salidroside pretreatment on expression of tumor necrosis factor- alpha and permeability of blood brain barrier in rat model of focal cerebralischemia-reperfusion injury. *Asian Pacific Journal of Tropical Medicine*.

[33] Wang C., Wang Q., Lou Y. (2017). Salidroside attenuates neuroinflammation and improves functional recovery after spinal cord injury through microglia polarization regulation. *Journal of Cellular and Molecular Medicine*.

[34] Su Y., Zong S., Wei C. (2018). Salidroside promotes rat spinal cord injury recovery by inhibiting inflammatory cytokine expression and NF‐*κ*B and MAPK signaling pathways. *Journal of Cellular Physiology*.

[35] Feng J., Zhang Q., Mo W. (2017). Salidroside pretreatment attenuates apoptosis and autophagy during hepatic ischemia-reperfusion injury by inhibiting the mitogen-activated protein kinase pathway in mice. *Drug Design, Development and Therapy*.

[36] Cai L., Li Y., Zhang Q. (2017). Salidroside protects rat liver against ischemia/reperfusion injury by regulating the GSK-3*β*/Nrf2-dependent antioxidant response and mitochondrial permeability transition. *European Journal of Pharmacology*.

[37] Chang X., Zhang K., Zhou R. (2016). Cardioprotective effects of salidroside on myocardial ischemia-reperfusion injury in coronary artery occlusion-induced rats and Langendorff-perfused rat hearts. *International Journal of Cardiology*.

[38] Chen J., Wang Q., Zhou W. (2018). GPCR kinase 2-interacting protein-1 protects against ischemia-reperfusion injury of the spinal cord by modulating ASK1/JNK/p38 signaling. *The FASEB Journal*.

[39] Lang-Lazdunski L., Matsushita K., Hirt L., Waeber C., Vonsattel J. P. G., Moskowitz M. A. (2000). Spinal cord Ischemia. *Stroke*.

[40] Chen X., Wu Y., Yang T. (2016). Salidroside alleviates cachexia symptoms in mouse models of cancer cachexia via activating mTOR signalling. *Journal of Cachexia, Sarcopenia and Muscle*.

[41] Takei N., Numakawa T., Kozaki S. (1998). Brain-derived neurotrophic factor induces rapid and transient release of glutamate through the non-exocytotic pathway from cortical neurons. *The Journal of Biological Chemistry*.

[42] Fu J., Sun H., Zhang Y. (2018). Neuroprotective effects of luteolin against spinal cord ischemia-reperfusion injury by attenuation of oxidative stress, inflammation, and apoptosis. *Journal of Medicinal Food*.

[43] Basso D. M., Fisher L. C., Anderson A. J., Jakeman L. B., Mctigue D. M., Popovich P. G. (2006). Basso mouse scale for locomotion detects differences in recovery after spinal cord injury in five common mouse strains. *Journal of Neurotrauma*.

[44] Rivlin A. S., Tator C. H. (1977). Objective clinical assessment of motor function after experimental spinal cord injury in the rat. *Journal of Neurosurgery*.

[45] Perrin F. E., Boniface G., Serguera C. (2010). Grafted human embryonic progenitors expressing neurogenin-2 stimulate axonal sprouting and improve motor recovery after severe spinal cord injury. *PLoS One*.

[46] Goldberg M. P., Choi D. W. (1993). Combined oxygen and glucose deprivation in cortical cell culture: calcium-dependent and calcium-independent mechanisms of neuronal injury. *The Journal of Neuroscience*.

[47] Fan J., Liu Y., Yin J. (2016). Oxygen-glucose-deprivation/reoxygenation-induced autophagic cell death depends on JNK-mediated phosphorylation of Bcl-2. *Cellular Physiology and Biochemistry*.

[48] Muraoka-Cook R. S., Caskey L. S., Sandahl M. A. (2006). Heregulin-dependent delay in mitotic progression requires HER4 and BRCA1. *Molecular and Cellular Biology*.

[49] Nicholson D. W., Thornberry N. A. (1997). Caspases: killer proteases. *Trends in Biochemical Sciences*.

[50] Yin X. H., Yan J. Z., Hou X. Y., Wu S. L., Zhang G. Y. (2013). Neuroprotection of S-nitrosoglutathione against ischemic injury by down- regulating Fas S-nitrosylation and downstream signaling. *Neuroscience*.

[51] Brentnall M., Rodriguez-Menocal L., De Guevara R., Cepero E., Boise L. H. (2013). Caspase-9, caspase-3 and caspase-7 have distinct roles during intrinsic apoptosis. *BMC Cell Biology*.

[52] Springer J. E., Azbill R. D., Knapp P. E. (1999). Activation of the caspase-3 apoptotic cascade in traumatic spinal cord injury. *Nature Medicine*.

[53] Qiu J., Nesic O., Ye Z. (2001). Bcl-x_L_ Expression after contusion to the rat spinal cord. *Journal of Neurotrauma*.

[54] Zhao L., Zhai M., Yang X. (2019). Dexmedetomidine attenuates neuronal injury after spinal cord ischaemia-reperfusion injury by targeting the CNPY2-endoplasmic reticulum stress signalling. *Journal of Cellular and Molecular Medicine*.

[55] Lazarou M., Sliter D. A., Kane L. A. (2015). The ubiquitin kinase PINK1 recruits autophagy receptors to induce mitophagy. *Nature*.

[56] Gladkova C., Maslen S. L., Skehel J. M., Komander D. (2018). Mechanism of parkin activation by PINK1. *Nature*.

[57] Koyano F., Okatsu K., Kosako H. (2014). Ubiquitin is phosphorylated by PINK1 to activate parkin. *Nature*.

[58] Matsuda N. (2016). Phospho-ubiquitin: upending the PINK-Parkin-ubiquitin cascade. *Journal of Biochemistry*.

[59] Carloni S., Buonocore G., Balduini W. (2008). Protective role of autophagy in neonatal hypoxia-ischemia induced brain injury. *Neurobiology of Disease*.

[60] Cassidy-Stone A., Chipuk J. E., Ingerman E. (2008). Chemical inhibition of the mitochondrial division dynamin reveals its role in Bax/Bak-dependent mitochondrial outer membrane permeabilization. *Developmental Cell*.

[61] Park S. W., Kim K. Y., Lindsey J. D. (2011). A selective inhibitor of drp1, mdivi-1, increases retinal ganglion cell survival in acute ischemic mouse retina. *Investigative Ophthalmology & Visual Science*.

[62] Yao N., Wang C., Hu N. (2019). Inhibition of PINK1/Parkin-dependent mitophagy sensitizes multidrug- resistant cancer cells to B5G1, a new betulinic acid analog. *Cell Death & Disease*.

[63] Bin-Umer M. A., McLaughlin J. E., Butterly M. S., McCormick S., Tumer N. E. (2014). Elimination of damaged mitochondria through mitophagy reduces mitochondrial oxidative stress and increases tolerance to trichothecenes. *Proceedings of the National Academy of Sciences of the United States of America*.

[64] Jiang X., Shi E., Nakajima Y., Sato S. (2006). Postconditioning, a series of brief interruptions of early reperfusion, prevents neurologic injury after spinal cord ischemia. *Annals of Surgery*.

[65] Guerit J. M., Dion R. A. (2002). State-of-the-art of neuromonitoring for prevention of immediate and delayed paraplegia in thoracic and thoracoabdominal aorta surgery. *The Annals of thoracic surgery*.

[66] Conrad M. F., Ye J. Y., Chung T. K., Davison J. K., Cambria R. P. (2008). Spinal cord complications after thoracic aortic surgery: long-term survival and functional status varies with deficit severity. *Journal of Vascular Surgery*.

[67] Greenlund L. J. S., Deckwerth T. L., Johnson E. M. (1995). Superoxide dismutase delays neuronal apoptosis: a role for reactive oxygen species in programmed neuronal death. *Neuron*.

[68] Skulachev V. P. (1996). Role of uncoupled and non-coupled oxidations in maintenance of safely low levels of oxygen and its one-electron reductants. *Quarterly Reviews of Biophysics*.

[69] Skulachev V. P. (1998). Cytochrome *c* in the apoptotic and antioxidant cascades. *FEBS Letters*.

[70] Jiang Y.-P., Ye R.-J., Yang J.-M. (2020). Protective effects of Salidroside on spermatogenesis in streptozotocin induced type-1 diabetic male mice by inhibiting oxidative stress mediated blood-testis barrier damage. *Chemico-Biological Interactions*.

[71] Huang Z., Fang Q., Ma W. (2019). Skeletal muscle atrophy was alleviated by salidroside through suppressing oxidative stress and inflammation during denervation. *Frontiers in Pharmacology*.

[72] Lin S. Y., Dan X., du X. X. (2019). Protective effects of salidroside against carbon tetrachloride (CCl_4_)-induced liver injury by initiating mitochondria to resist oxidative stress in mice. *International journal of molecular sciences*.

[73] Feng J., Chen X., Guan B., Li C., Qiu J., Shen J. (2018). Inhibition of peroxynitrite-induced mitophagy activation attenuates cerebral ischemia-reperfusion injury. *Molecular Neurobiology*.

[74] Zhang Z., Xu T., Chen J. (2018). Parkin-mediated mitophagy as a potential therapeutic target for intervertebral disc degeneration. *Cell Death & Disease*.

[75] Larsen K. E., Sulzer D. (2002). Autophagy in neurons: a review. *Histology and histopathology*.

[76] Bell M. T., Puskas F., Agoston V. A. (2013). Toll-like receptor 4-dependent microglial activation mediates spinal cord ischemia-reperfusion injury. *Circulation*.

[77] Zhu P., Li J. X., Fujino M., Zhuang J., Li X. K. (2013). Development and treatments of inflammatory cells and cytokines in spinal cord ischemia-reperfusion injury. *Mediators of Inflammation*.

[78] Wang L., Yao Y., He R. (2017). Methane ameliorates spinal cord ischemia-reperfusion injury in rats: antioxidant, anti-inflammatory and anti-apoptotic activity mediated by Nrf2 activation. *Free Radical Biology & Medicine*.

[79] Gökce E. C., Kahveci R., Gökce A. (2016). Neuroprotective effects of thymoquinone against spinal cord ischemia-reperfusion injury by attenuation of inflammation, oxidative stress, and apoptosis. *Journal of Neurosurgery. Spine*.

[80] Liu X., Wen S., Yan F. (2018). Salidroside provides neuroprotection by modulating microglial polarization after cerebral ischemia. *Journal of Neuroinflammation*.

