# Supplementary figures and images for: Salidroside Ameliorates Mitochondria-Dependent Neuronal Apoptosis after Spinal Cord Ischemia-Reperfusion Injury Partially through Inhibiting Oxidative Stress and Promoting Mitophagy
Source: Oxid Med Cell Longev. 2020 Jul 23;2020:3549704. doi: 10.1155/2020/3549704 (PMC7396093; doi:10.1155/2020/3549704)

Supplementary material

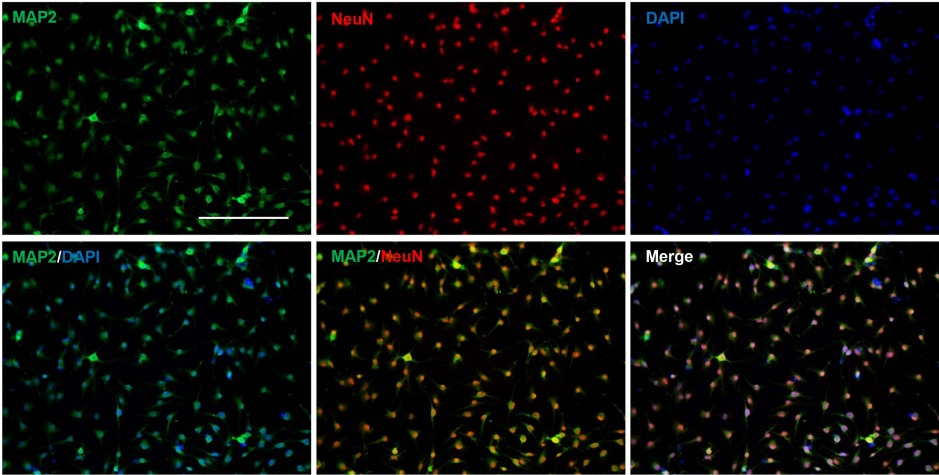

Supplement: Supplementary Materials — Immunocytochemical identification of primary mouse spinal cord neurons. Neuronal dendrites and axons were identified by anti-MAP2 (green) and somata by NeuN (red) immunostaining. The nuclei of all cells were identified by DAPI (blue). Scale bars, 100 μm. Neuronal cultures with cell purity greater than 90 percent were used in this study. [file 3549704.f1.pdf]
